# Multi-Depot Open Vehicle Routing Problem with Time Windows Based on Carbon Trading

**DOI:** 10.3390/ijerph15092025

**Published:** 2018-09-17

**Authors:** Ling Shen, Fengming Tao, Songyi Wang

**Affiliations:** 1 College of Mechanical Engineering, Chongqing University, Chongqing 400044, China; shenlingling@cqu.edu.cn (L.S.); songyi_wang@cqu.edu.cn (S.W.); 2 School of Economics and Business Administration, Chongqing University, Chongqing 400044, China

**Keywords:** open vehicle routing problem, particle swarm optimization algorithm, tabu search algorithm, carbon trading prices, carbon quotas

## Abstract

In order to cut the costs of third-party logistics companies and respond to the Chinese government’s low-carbon economy plans, this paper studies the more practical and complex open vehicle routing problem, which considers low-carbon trading policies. A low-carbon multi-depot open vehicle routing problem with time windows (MDOVRPTW) model is constructed with minimum total costs, which include the driver’s salary, penalty costs, fuel costs and carbon emissions trading costs. Then, a two-phase algorithm is proposed to handle the model. In the first phase, the initial local solution is obtained with particle swarm optimization (PSO); in the second phase, we can obtain a global optimal solution through a further tabu search (TS). Experiments proved that the proposed algorithm is more suitable for small-scale cases. Furthermore, a series of experiments with different values of carbon prices and carbon quotas are conducted. The results of the study indicate that, as carbon trading prices and carbon quotas change, total costs, carbon emission trading costs and carbon emissions are affected accordingly. Based on these academic results, this paper presents some effective proposals for the government’s carbon trading policy-making and also for logistics companies to have better route planning under carbon emission constraints.

## 1. Introduction

Rapid economic growth is accompanied by high energy consumption, resulting in large amounts of carbon emissions [1]. This has accelerated the pace of global warming and has become a significant choke point for sustainable development. As we all know, China is a large energy consumption country which has plenty of carbon emissions and they are always trying to solve it. In 2005, the Chinese government made a promise to reduce carbon dioxide emissions per unit of GDP by 40–45% by 2020 [2]. China is setting up a national carbon trading market in 2018. Carbon dioxide is the main source of greenhouse gases [3], the emissions of which result from human activities, for example the fossil fuel combustion in logistics. According to statistics, transportation accounted for 14% of global carbon emissions and road transport represented almost three-quarters of these emissions [4,5]. Therefore, the low-carbon logistics industry is important for achieving a low-carbon economy. Carbon trading is an effective and important tool for reducing carbon emissions [6].

**Figure 1:**
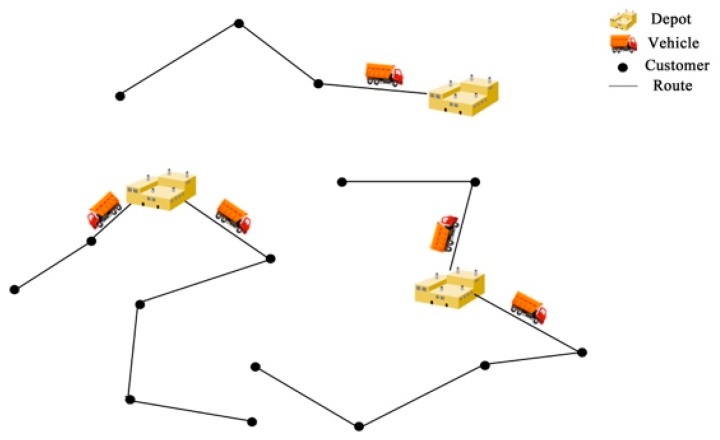
A simplified diagram of the multi-depot open vehicle routing problem time window (MDOVRPTW).

The open vehicle routing problem (OVRP) is that vehicles do not return to the depot [7]. In reality, transportation problems include some constraints. The open vehicle routing problem with time window (OVRPTW) adds the time window restrictions to meet the customer’s demand. Instead of the single-depot problem, the multi-depot problem is proposed in the multi-depot open vehicle routing problem (MDOVRP). In real-life routing applications, time window and multi-depot constraints usually exist in the OVRP at the same time, which is shown in Figure 1 and called the multi-depot open vehicle routing problem time window (MDOVRPTW). Due to study from time and space, it is more complicated than other kinds of transportation problems. The MDOVRPTW problem has important application value in real life. For example, if the company owns multiple parking lots and the vehicle does not need to come back to the original departure depot after accomplishing the task, it can save the company’s overall transportation costs. The vehicle can both increase customer contentment and reduce the penalty cost due to the early or late arrival of the vehicle if it can complete the distribution task within the time window specified by the customer.

This paper is organized as follows: a literature review of related work is presented in Section 2; the problem and the model formulation are proposed in Section 3; a two-phase algorithm is described in Section 4; Section 5 discusses the computational results; and finally, Section 6 presents the conclusions.

## 2. Literature Review

As the core thought of the current research is to obtain a better solution for MDOVRPTW, considering carbon trading. We review the literature in two areas: algorithm of open vehicle routing problem and objective function considering carbon emissions.

### 2.1. Algorithm of the Open Vehicle Routing Problem

OVRP was first addressed by Schrage [7] in 1981. Since then, there has emerged much research into a variety of OVRPs and a number of studies have been conducted into its modeling and solutions, such as the Branch-and-Cut-and-Price method [8], the ant colony optimization algorithm [9], the particle swarm optimization algorithm [10], the variable neighborhood search algorithm [11], the tailored Iterated Local Search algorithm [12], the tabu search heuristic algorithm [13], and so forth.

Repoussis et al. [14] first researched the OVRPTW. Brandão [15] proposed an iterated local search algorithm for dealing with the OVRPTW, which was able to obtain a highly quality solution within a reasonable computing time. Recently, Niu et al. [16] studied green OVRPTW, which was optimized by a tabu search heuristic algorithm. The neighborhood search strategies were adopted to improve this algorithm. MDOVRP was first presented by Tarantilis and Kiranoudis [17] to deal with the fresh meat, which is a real-life distribution problem. In the study, a list-based threshold accepting algorithm was used. For the MDOVRP, an effective meta-heuristic solution approach—termed the hybrid genetic algorithm—was proposed by Liu et al. [18]. They presented a mixed integer programming (MIP) mathematical formulation to handle this problem. Then, Lalla-Ruiz et al. [19] presented a new mixed integer programming mathematical constitution, which outperformed the previous study of Liu et al. [18]. Some new restrictions were introduced in this research. Soto et al. [20] developed a multiple variable neighborhood search, hybridized with a tabu search (MNS-TS), to deal with the MDOVRP. In this article, authors compared MNS-TS with MIP and found that the average quality of the solutions was different. In 2008, Duan [21] suggested a better tabu search algorithm, which was improved by the new neighborhood transformation. The high-quality vehicle routing optimization solution was quickly obtained through the example test which has strong and direct applicability to the logistics distribution practice. In 2013, Liu and Ma [22] addressed the hybrid genetic algorithm, which linked the Clarke and Wright saving algorithm with the scanning algorithm. Considering the lack of business operation, vehicle leasing and sharing were added into the MDOVRPTW in the paper. In 2017, Ling et al. [23] carried out the optimal solution using the new ant colony algorithm, which was optimized by the 2-optimization algorithm. The proposed method was effective for the MDOVRPTW, which was verified by the experimental results.

### 2.2. Objective Function Considering Carbon Emissions

Most of the research objectives of these vehicle routing problems outlined above were only focused on economic efficiency. With more concern for environmental issues, many scholars began to add energy and environmental protection costs to the objective function [24]. Zhang et al. [25] presented the evolutionary local search algorithm to solve the special variant of the united loading and routing problem. Rather than minimizing travel distances, the objective of the paper was to minimize vehicle fuel consumption. Li et al. [26] revealed the new short-term tactical problem and proposed the objective of minimizing carbon dioxide emissions per ton kilometer. Later, a further study about the complex emission effected by many factors was presented by Naderipour and Alinaghian [27]. They used the MEET model, which was first introduced by Hickman [28]. The target of the paper was to minimize costs, which include driver wages and emission costs. Similarly, Gao and Liu [29] proposed the cost function including fuel costs and driver costs. But their research was about the time-dependent pollution-routing problem which has the free airport service for pickup and delivery of customers. Zhang et al. [30] built a low-carbon routing problem model. They put fuel consumption costs, carbon emissions costs and vehicle usage costs into the conventional VRP problem as the new objective function. Liao [31] proposed the minimizing costs related to economics and emissions for the on-line vehicle routing problem. Then, Niu et al. [16] investigated the third-party logistics to minimize the routing costs which include fuel costs, carbon dioxide emissions costs and driver costs. This study is part of the green OVRPTW. Duan and He [32] studied the multiple depots incomplete OVRP with soft windows considering carbon tax. This paper found that both profit and carbon emissions were improved despite the carbon tax. Recently, Wang et al. [5] proposed a location routing problem model, which is green and low-carbon. The objective function of this study is the minimum overall costs, which considers carbon emissions. In order to analyze the influence of the carbon tax on the carbon emissions and total costs, carbon tax policies are recommended in this paper.

In brief, based on the above analysis, there are many studies on the open vehicle routing problem’s variants algorithm. Meanwhile, research on the objectives of vehicle routing problems considering carbon emissions is abundant. Nevertheless, there is little literature that considers carbon emissions with regard to the MDOVRPSTW. The above literature [32] is the only close-related one. In view of this, we propose the algorithm and objective function of this paper as follows: (1) This paper uses a two-phase algorithm to handle the MDOVRPSTW based on carbon trading. The PSO is used to get the initial solution, which has high efficiency for OVRP [11,33]. The TS is used to get the global optimal solution, which has the global solution for OVRP [10,13,34]. The high-quality vehicle routing optimization solution is quickly obtained for the small-scale cases, which has strong and direct applicability to the logistics distribution practice; and (2) This paper minimizes the overall costs from the three perspectives: economical costs, energy-related costs and environmental protection costs. The economic costs of the entire distribution network are penalty costs and drivers’ salary costs. The energy costs are fuel consumption costs. The environmental protection costs are carbon emission trading costs, which are related to the actual carbon emissions and total carbon emission limits of the logistics network.

## 3. Model Formulation

### 3.1. Problem Description

The government takes various measures to deal with the development of a low-carbon economy and logistics. This topic attracts much research attention. There is currently a lot of literature available on carbon emission trading. Against this background, this paper studies the MDOVRPTWCT based on the perspective of third-party logistics companies. CO_2_ is considered the only greenhouse gas in this study.

This problem can be described as follows: There are *D* (*d* = 1, 2, …, *d*) depots with vehicles provided by third-party transportation service providers. The total number of vehicles provided is fixed as *H*. Vehicles begin from the distribution center to service customers, then end at the last customer, which means vehicles need not return to the depot. Each customer can be served only once by exactly one vehicle. In fact, customers always have some request about the delivery time. The vehicle must pay the penalty if it is earlier or later than the arrival time window. The vehicle capacity and customer demand are foregone and the demand of customers on each route cannot overtake the capacity of vehicle. What is more, the feasible route must satisfy the route length constraints, which means each route has an upper limit for the total traveling time. The objective of this research is mainly to arrange transport routes and to explore the effects of carbon trading on total costs and carbon emissions.

### 3.2. Symbols

Based on the demands of structuring the model, Table 1 shows the corresponding symbols adopted in this paper.

### 3.3. Model Development

In this section, we first introduce the sub-cost of this optimization in detail, then present the specific formulation of the MDOVRPTW based on carbon trading.

#### 3.3.1. Objective Function Analysis

##### Drivers’ Salary Costs

When the vehicles of the third-party logistics company’s depot are not enough, other companies’ vehicles can be rented. In this article, each rental vehicle is equipped with a driver and it must pay a fixed salary to the driver. When the number of vehicles actually used is less than all vehicles, there is no need to rent vehicles. Hence, the drivers’ salary costs $C_{1}$ are 0. Otherwise, it can be calculated with the equation:

(1) $$C_{1}=C_{v}\left(\overset{h_{d}}{\underset{h=1}{\sum }}\overset{d}{\underset{i=1}{\sum }}\overset{d+c}{\underset{j=d+1}{\sum }}x_{ij}^{dh}-H\right)$$

##### Penalty Costs

In the actual distribution, due to the customer’s time requirements, the vehicle routing problem with time window has been much researched. According to the strictness of time constraints, the time window is usually divided into two categories: hard time window [35] and soft time window [36]. The time when vehicle *h* reaches the customer *i* can be expressed as:

(2) $$T_{i}^{h}=T_{i}^{h}+s_{i}+c_{ij}.$$

This paper studies the soft time window, proposing the penalty costs $C_{2}$, which can be calculated as:

(3) $$C_{2}=\overset{h_{d}}{\underset{h=1}{\sum }}\overset{d+c}{\underset{i=d+1}{\sum }}(C_{p1}max\left\{ET_{i}-T_{i}^{h},0\right\}+C_{p2}max\left\{T_{i}^{h}-LT_{i},0\right\}).$$

##### Fuel Consumption Costs

In the current situation, to calculate the fuel consumption of a vehicle is complicated, because it is affected by many factors such as: car characteristics, road traffic conditions, road gradient, driving level and so forth, thus, it is too difficult and unnecessary to take all factors into consideration. Thus, we refer to the literature [5] to calculate fuel consumption. The total fuel consumption costs $C_{3}$ are expressed as follow:

(4) $$C_{3}=\overset{h_{d}}{\underset{h=1}{\sum }}\overset{d+c}{\underset{i=1}{\sum }}\overset{d+c}{\underset{j=1}{\sum }}C_{e}\left(r+\frac{r^{\prime }-r}{Q}Q_{i}^{\prime }\right).$$

##### Carbon Emission Trading Costs

The literature has shown that the carbon emissions of vehicles have a relationship with fuel consumption. This paper uses the following Formula (5) to calculate the amount of carbon dioxide emissions from fuel consumption. Table 2 presents some data related to carbon dioxide emissions.

(5) $$T_{CO_{2}}=\eta F_{fuel}.$$

Next, we need to introduce carbon quotas and carbon subsidies. When the actual carbon emissions are lower than the total amount of carbon emissions, companies can sell carbon emission rights to gain profit. If the carbon emissions are greater than the upper limit, the company must purchase additional carbon subsidies [38]. It is assumed that the carbon emissions quotas are $T_{q}$ and the carbon trading price is $C_{ctp}$. Therefore, the carbon emission trading costs $C_{4}$ can be calculated as follows:

(6) $$C_{4}=C_{ctp}\left(\eta F_{fuel}-T_{q}\right)$$

#### 3.3.2. Model Setting

On the basis of the above analysis, the mathematical model built in this study is expressed as follows:

(7) $$\begin{array}{c} \min F=C_{1}+\overset{h_{d}}{\underset{h=1}{\sum }}\overset{d+c}{\underset{i=d+1}{\sum }}(C_{p1}max\left\{ET_{i}-T_{i}^{h},0\right\}\\ \;+C_{p2}max\left\{T_{i}^{h}-LT_{i},0\right\})\\ \;+\overset{h_{d}}{\underset{h=1}{\sum }}\overset{d+c}{\underset{i=1}{\sum }}\overset{d+c}{\underset{j=1}{\sum }}C_{e}\left(r+\frac{r^{\prime }-r}{Q}Q_{i}^{\prime }\right)+C_{ctp}\left(\eta F_{fuel}-T_{q}\right) \end{array}$$

Subject to:

(8) $$\overset{h_{d}}{\underset{h=1}{\sum }}\overset{d}{\underset{d=1}{\sum }}\overset{d+c}{\underset{i=1}{\sum }}x_{ij}^{dh}=1,j\in \left\{d+1,d+2,\ldots ,d+c\right\}$$

(9) $$\overset{d}{\underset{d=1}{\sum }}\overset{d+c}{\underset{i=d+1}{\sum }}\overset{d}{\underset{j=1}{\sum }}x_{ij}^{dh}=1,\forall h\in \left\{1,2,\ldots ,H^{\prime }\right\}$$

(10) $$\overset{d}{\underset{d=1}{\sum }}\overset{d+c}{\underset{i=d+1}{\sum }}\overset{d+c}{\underset{j=d+1}{\sum }}x_{ij}^{dh}=1,\forall h\in \left\{1,2,\ldots ,H^{\prime }\right\}$$

(11) $$\overset{d}{\underset{d=1}{\sum }}\overset{d}{\underset{i=1}{\sum }}\overset{d}{\underset{j=1}{\sum }}x_{ij}^{dh}=0,\forall h\in \left\{1,2,\ldots ,H^{\prime }\right\}$$

(12) $$\overset{h_{d}}{\underset{h=1}{\sum }}\overset{d}{\underset{d=1}{\sum }}\overset{d+c}{\underset{i=1}{\sum }}\overset{d+c}{\underset{j=d+1}{\sum }}q_{i}x_{ij}^{dh}\leq Q_{0},\forall h\in \left\{1,2,\ldots ,H^{\prime }\right\}$$

(13) $$\overset{h_{d}}{\underset{h=1}{\sum }}\overset{d}{\underset{d=1}{\sum }}\overset{d+c}{\underset{i=1}{\sum }}\overset{d+c}{\underset{j=d+1}{\sum }}\left(c_{ij}+s_{i}\right)x_{ij}^{dh}\leq L,\forall h\in \left\{1,2,\ldots ,H^{\prime }\right\}$$

(14) $$\overset{d}{\underset{d=1}{\sum }}\underset{i\in G}{\sum }\underset{j\in G}{\sum }x_{ij}^{dh}\leq \left|G\right|-1,G\in C,2\leq \left|G\right|\leq c,\forall h\in \left\{1,2,\ldots ,H^{\prime }\right\}$$

The objective function (7) is to minimize the total costs of the third-party logistics system, which includes four sub-costs: drivers’ salary costs, penalty costs, fuel consumption costs and carbon emission costs. Constraint (8) shows that each customer must be serviced once by a vehicle. A vehicle begins at the depot and ends at the last visited customer, which is imposed by Constraints (9) and (10). Constraint (11) defines that vehicles cannot go directly from the depot to the depot. Constraint (12) represents that the needs of customers for each path do not pass the maximum load of the vehicle. The total time for each path does not exceed the longest length of the route, which is provided by Constraint (13). Constraint (14) illustrates the subtour elimination.

## 4. A Two-Phase Algorithm

OVRP is a typical NP-hard problem [13,39], which can be solved by various kinds of heuristics. The PSO algorithm is simple and flexible [40] but it usually gets in local optimum. Tabu search can avoid roundabout searching. Hence, in this paper, the improved particle swarm optimization algorithm with tabu search is advanced to deal with the mathematical model above. The proposed algorithm is composed of two phases [41]. First, a particle swarm algorithm is adopted to generate an initial optimal solution. Second, a tabu search is used to optimize the initial optimal solution to make it jump out of the local optimum.

The PSO algorithm originates in the parallel evolutionary computation technique, which was developed by Kennedy and Eberhart [38], according to the social behavior metaphor. It is paid great attention owing to its good performance on memory occupation and calculation speed. The PSO algorithm is widely used in many fields, such as: combination optimization, fuzzy system control, function optimization and so on, but this algorithm may turn out to be premature. Tabu search is an intelligent heuristic global neighborhood search algorithm. It simulates the human search feature with memory function. This paper mainly adopts the idea of jumping away from the local optimum, using the medium-term and long-term memory methods to improve the particle swarm optimization algorithm.

### 4.1. Encoding and Decoding

The particle swarm optimization algorithm has a main problem, which is how the position of the particle corresponds to the solution of the model. And it is common to use the 3*n*-dimensional vector for the multi-depot problem. This article constructs an indirect coding method for the particle, according to the literature [42], which is based on parking lot and customer arrangements. It essentially transforms multi-depot issues into a single depot. This encoding method has the following two advantages: (1) The code length is fixed, which is equal to *d* + *c* − 1 (*d* is the number of depots and *c* is the number of customers) and easy to compute; and (2) It does not pre-estimate the number of vehicles used. The latitude *X* of the particle is the random numbers related to depot and customer information.

**Figure 2:**
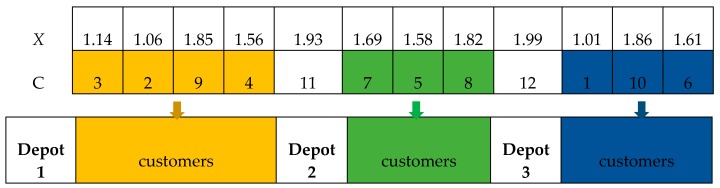
Allocation of depots and customers.

The first step is to allocate customers to different depots. Firstly, it needs to sort the latitude *X* of the particle and find the serial number *C* of the corresponding location. Then, finding the positions with the serial number greater than the number of customers; these represent the depot’s information and the other represents the customer’s information. For example, supposing there are 10 customers and 3 depots. The detailed information is shown in Figure 2.

**Figure 3:**
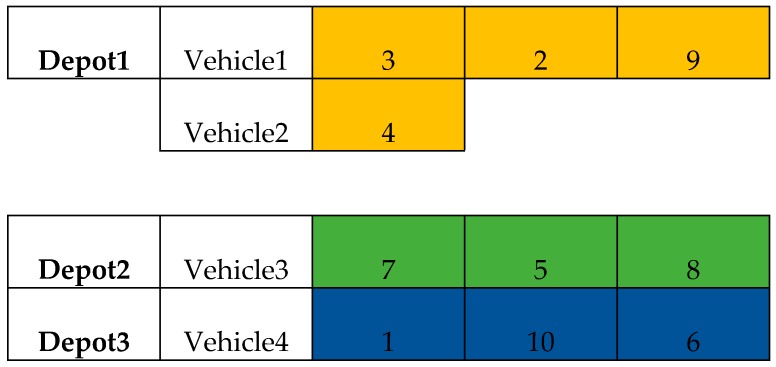
Detailed solution routing in each depot.

The second step is to route the customers inside the depot. The routing division in each depot is essentially a vehicle routing optimization problem in a single depot. The single-site vehicle routing encoding method is as follows: Each node is considered in sequence until the node demand, during time and so forth, violates the constraint. At this time, the previously checked node is taken as a routing and the remaining nodes are continuously checked until all the nodes in the site are divided. For the above example, the maximum time of each path is 10 and the maximum load per vehicle is 30. The start time is 0. Table 3 shows the customers’ demand, time information. The time here is the last node to the customer’s time. Then the corresponding solution routing is shown in Figure 3.

The encoding and decoding of this particle can ensure that each customer is served once and that each customer can be limited to only one vehicle. What is more, the calculation of the solution process can be reduced.

### 4.2. Fitness Function

Since the path has satisfied the vehicle capacity and vehicle time constraints in the course of particle decoding, the fitness function of this paper can be expressed as:$\;Fitness\left(X_{i}\right)=F$.

### 4.3. Constructing Initial Optimal Solution Based on PSO Algorithm

In the first phase, we use the PSO algorithm to obtain a high-quality initial solution. When the algorithm starts, each solution in the solution space is considered as a particle. In the shrinking space, each particle has a position to determine its position and a speed to determine its distance and direction.

#### 4.3.1. Initialization

Parameter initialization, set the length of particle code *VarSize*, the number of population *nPop*, maximum number of iterations *MaxIt*, inertia weight *W*, the number of *R*_1_, *R*_2_, acceleration factor *C*_1_, *C*_2_ and the particle range [*VarMin*, *VarMax*] and velocity range [−0.1*(*VarMax*−*VarMax*), 0.1*(*VarMax*−*VarMax*)]. The solution vector is a 1-dimensional variable in this paper. When the particles are initialized, the position and velocity of the ith population can be expressed as:

(15) $$\begin{array}{c} X_{i}=rand\left(VarSize\right).*\left(VarMax-VarMin\right)+VarMin,\\ V_{i}=rand\left(VarSize\right).*\left(VelMax-VelMin\right)+VarMin. \end{array}$$

#### 4.3.2. Determining Optimal Solution

All particles have a fitness value that is determined by the fitness function. In each iteration, each particle has an individual extremum and all particles share a global extremum. Particles follow the individual extremes and global extremes to search in the solution space and find the optimal solution. The individual extremum is expressed as *P^best^* and the common global extremum of all particles is denoted as *G^best^*.

#### 4.3.3. Particle Status Update

Each time the position and velocity are updated according to the following formulas:

(16) $$\{\begin{array}{c} V_{i+1}=WV_{i}+C_{1}R_{1}\left(P^{best}-X_{i}\right)+C_{2}R_{2}\left(G^{best}-X_{i}\right),\\ if\;V_{i+1}\;>V_{max},\;V_{i+1}\;=V_{max},\\ if\;V_{i+1}\;<V_{min},\;V_{i+1}\;=V_{min},\\ X_{i+1}=X_{i}+V_{i+1}. \end{array}$$

These formulas respectively reflect the memory of particle, the particle’s thinking and information sharing and mutual cooperation between particles.

#### 4.3.4. Terminating Condition

Finally, when the greatest population number *nPop* appears is the end of the condition. Otherwise, it will continue to evolve.

### 4.4. Structuring Global Optimal Solution Based on TS

#### 4.4.1. Initial Solution

In the second phase, the solution is further optimized by the tabu search. For the initial solution, we adopt the optimal solution in the first phase. Initialize tabu list, determining the tabu length and so on.

#### 4.4.2. Neighborhood Structure Design

The tabu search algorithm is an algorithm of the neighborhood search technology and determining the neighborhood operation is a major procedure in composing the algorithm. According to the characteristics of path encoding and decoding in this paper, this paper selects three routes for the neighborhood search algorithm: swap, reversion, insertion. In this study, we use the computer to generate a random number which is between 1 and 3. Then one of three operators is adopted at the same possibility according to the generated random number.

#### 4.4.3. Tabu Objects and Tabu Table

The tabu search algorithm leads the algorithm to hunt for areas that have not been searched in the problem space by means of tabu search solution improvement factors and retrieves previously searched solutions. In this paper, the component vectors of tabu solutions are used as tabu objects. This method can avoid repeated searches and save time. For three different search methods, three tabu tables are created to store the corresponding tabu objects. The tabu tabulation element *tabu_i_* (*i* = 1, 2, 3) marks the contraindication of the corresponding it need to adopt special rules to unlock the tabu candidate solution.

#### 4.4.4. Terminating Condition

To get the solution in a reasonable time, tabu search need the termination condition in the iterations. In this paper, we take the maximum number of iterations as the termination criteria.

### 4.5. Steps of PSO-TS Algorithm

**Figure 4:**
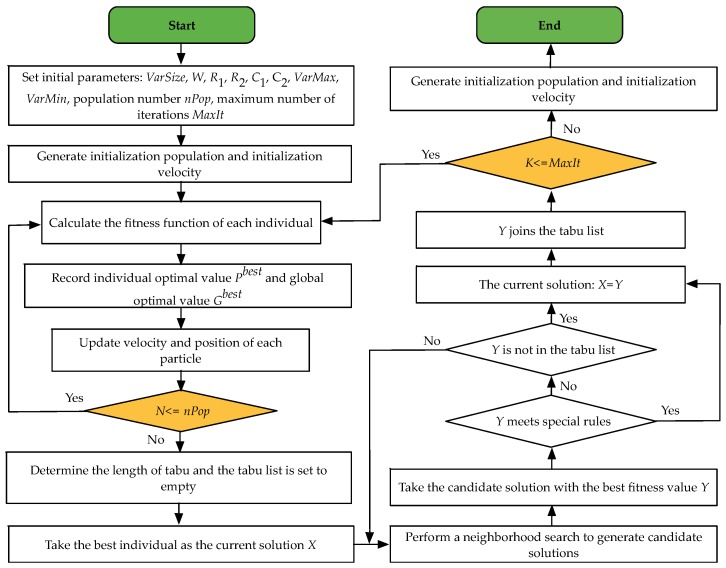
Particle swarm optimization-tabu search (PSO-TS) flowchart.

The TS-PSO algorithm detailed flowchart for solving the MDOVPRPTWLC model is shown in Figure 4.

## 5. Computational Experiments

### 5.1. Test Cases

The benchmark data sets of Cordeau [43] are well-known in MDVRPTW. In this study, we choose the narrow time window problem pro01–pro10 from the typical database. Then, they are applied to the MDOVRPTW and used to test the two-phase algorithm. The distance in the test instances is the Euclidean distance and supposing that between two nodes the travel time of the vehicle is equal to the Euclidean distance. This article uses the literature [44] classification method to divide the test cases into three categories according to the number of customers: small-scale study 1–100 (pro01, pro02 and pro07), medium-scale study 101–200 (pro03, pro04 and pro08), large-scale study 201–300(pro05, pro06, pro09 and pro10). Table 4 shows part of information about the test instances, which includes the number of customers *C*, depots *D*, vehicles *H*, maximum vehicle load *Q*_0_ and longest duration of each route *L*. And we set the parameters of this model according to the former studies [16,45,46], which are shown in Table 5. According the Table 2, η can be calculated as 2.623. We set the carbon trading price $C_{ctp}$ as 25 CNY/t, which refers to the literature [37].

### 5.2. Parameter Setting for PSO-TS

The parameter setting for the PSO-TS is very vital, which can affect the quality and efficiency of the results. We can obtain a better solution though the appropriate parameter setting. Based on previous studies [47,48] and a large number of experiments, the parameters are set in Table 6. The proposed algorithm in this paper is coded using programming language on MATLAB R2017a (MathWorks, Natick, Massachusetts, USA) and all the experiments are evaluated on a PC with Intel 2.3 GHz processor and 8GB RAM.

### 5.3. Effectiveness of Two-Phase Algorithm

As we all know, the carbon quota is generally set by the government. Here, we initially assume that the carbon quota is 0 and we set the carbon trading price as 0.025 CNY/kg. To investigate the effectiveness of the algorithm, we propose the traditional particle swarm optimization to compare with the proposed two-phase algorithm. For each of the following experiments, we perform them 20 times and record the best value as the optimal result. Table 7 shows the detailed computational results of PSO and PSO-TS, which include total costs, length of route, carbon emission and optimization rate of total costs.

We can easily see from Table 7, compared with PSO algorithm, total costs, length of route, carbon emissions of the PSO-TS algorithm all have a great improvement in the quality of solution. And the average optimization rate of total costs reaches to 35.21%.

In this paper, we propose three scale cases: small, medium and large and we calculate the ten cases (pro01–pro10) with the PSO-TS algorithm. Table 8 shows the detailed calculation results, which include vehicles’ number, drivers’ salary, penalty costs, fuel costs (fuel consumption costs), carbon costs (carbon emission trading costs), total costs, carbon emissions and length of route.

From the Table 8, we find the drivers’ salary of these cases (pro01, pro02 and pro07) are all zero and those cases all belong to small scale. We propose an assumption that the two-phase algorithm is more suitable for small-scale cases.

**Figure 5:**
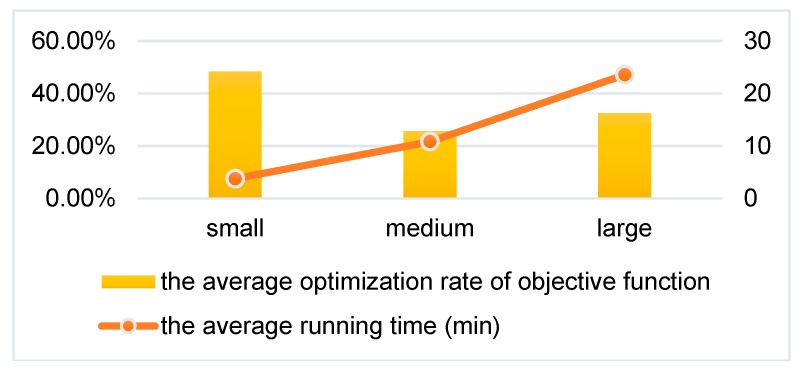
The average optimization rate and running time of three scale cases.

To verify the applicability of the PSO-TS algorithm, we calculate the average optimization rate of objective function and the average running time of the three scales. The results are shown in the Figure 5. The average running time demonstrates the computational complexity of the algorithm. The average optimization rate of objective function illustrates the performance of the algorithm.

It can be seen in Figure 5, with the increase in the size of cases, the PSO-TS algorithm needs to spend more time to get the optimal result and the best average optimization rate of objective function 43.73% is of the small-scale case. Considering the speed of convergence and the quality of solution, the proposed two-phase algorithm is more suitable for small-scale problems.

### 5.4. Experimental Results

In the carbon trading environment, carbon quotas and carbon trading prices have a direct bearing on environmental protection costs (carbon emission trading costs) and they can indirectly change the vehicle arrangements and route planning, which will further affect economic costs (penalty costs and drivers’ salary costs) and energy costs (fuel consumption costs). In the following, we conduct a detailed study on carbon trading prices and carbon quotas respectively.

In the following research, we only study the three cases (pro01, pro02 and pro07), because the proposed algorithm is more applicable to small-scale cases. What is more, we perform 20 times for each of the following experiments and record the best value as the optimal result. Then decoding the optimal solution can obtain total costs, each sub-cost, carbon emissions and so on.

First, in order to study the impact of carbon trading price on the objective function and carbon emission, we set up a comparative experiment for small-scale cases. Here, we just consider the carbon trading price, thus the carbon quotas are 0. We set the carbon trading price to 0, calculate the values associated with the objective function and compare it with the previous experiment results shown in Table 8 when the carbon trading price is 0.025. Table 9 shows the detailed values of carbon costs, fuel costs, total costs, carbon emissions when the carbon trading price is 0 and 0.025 respectively.

According to the results in Table 9, the following findings can be observed. When carbon trading prices are 0, the carbon costs are also 0. When carbon trading prices is 0.025, the carbon costs are bigger than 0. That the carbon trading price is 0 means there is not a carbon prices constraint. Compared with the case in which the carbon trading price is 0, the fuel costs and total costs of the three cases (pro01, pro02 and pro07) are all lower when carbon trading price is 0.025. In addition, carbon emissions of the three cases (pro01, pro02 and pro07) have also been effectively improved when carbon trading prices are 0.025. In conclusion, it shows that under the constraints of carbon trading prices, even if the carbon costs increase, the total costs as the objective function and fuel costs can reduce and the carbon emissions can also reduce.

Next, we study the effect of carbon quotas on the objective function and carbon emissions for fixed carbon trading prices. Here, the fixed carbon trading prices are 0.025. The carbon emissions quotas are difficult to estimate the exact value. In this study, for each case, we set 3 new carbon quotas ($T_{q}^{\prime }$) around the carbon emissions (pro01:615.50, pro02:1411.18, pro07:1208.70), which can be obtained from Table 8 when the carbon trading price is 0.025 and the carbon quota is 0. Aimed at the new different carbon quotas, the values of total costs, carbon costs, carbon emissions and the difference between $T_{q}^{\prime }$ and carbon emissions (CE) can be calculated respectively, which are shown in Table 10. 

According to the results in Table 10, the following findings can be observed. With the increase of carbon quotas, for each case, the total costs and carbon costs also increase. Since the carbon quotas are a small increase, the increase of total costs and carbon costs is slight. However, as the carbon quotas increase, the carbon emissions are a fixed value for each case (pro01:615.50, pro02:1411.18, pro07:1208.70). We know that the calculation of carbon costs is related to the difference between carbon quotas and carbon emissions. The increase in carbon costs is due to the increase in the difference between CE and $T_{q}^{\prime }$. For each case, the change of this difference ($T_{q}^{\prime }$−CE) can be seen from Table 10.

**Figure 6:**
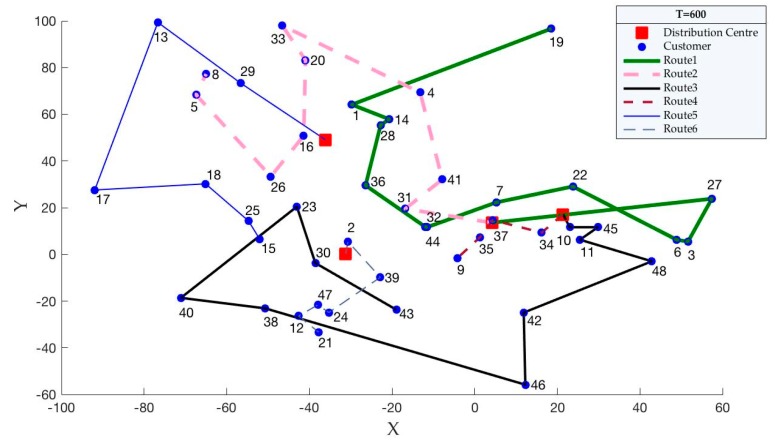
Distribution paths when $T_{q}^{\prime }$ are 600.

**Figure 7:**
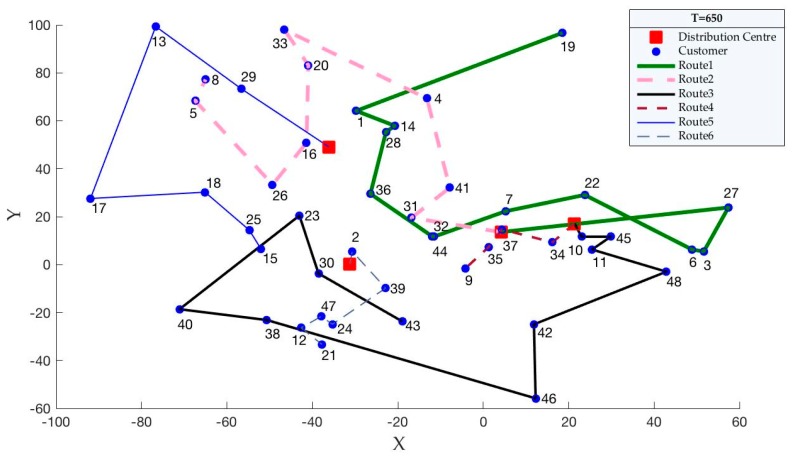
Distribution paths when $T_{q}^{\prime }$ are 650.

**Figure 8:**
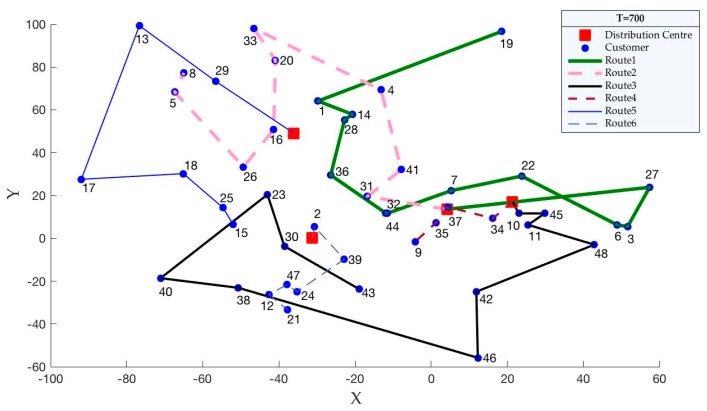
Distribution paths when $T_{q}^{\prime }$ are 700.

On the basis of the findings, when carbon quotas change, carbon emissions are the fixed value for each case. We know that carbon emissions are related to the distribution paths. Therefore, we propose an assumption that when the carbon quota changes, the distribution paths are unchanged. Then, we use the case of pro01 to verify this hypothesis. According to Table 10, we set the carbon quotas ($T_{q}^{\prime }$) as 600, 650 and 700. Then, the optimal distribution paths for pro01, which are obtained by solving the model, are shown in Figure 6, Figure 7 and Figure 8.

According to Figure 6, Figure 7 and Figure 8, we can easily see that the optimal distribution paths for pro01 are same when the carbon quotas are 600, 650 and 700. Therefore, the changed carbon quotas have no effect to the optimal distribution paths, which supports our hypothesis.

**Figure 9:**
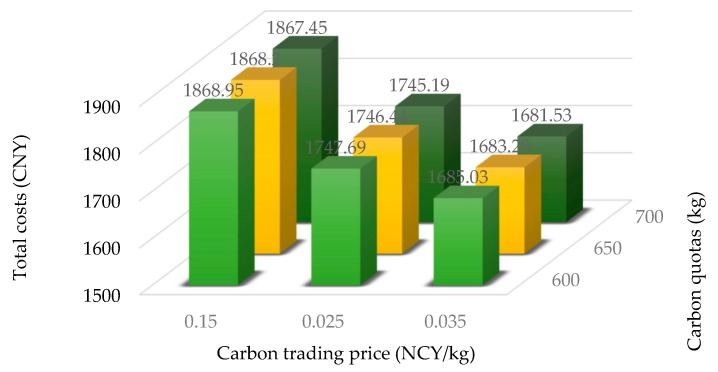
The total costs under different carbon trading price and carbon quotas.

**Figure 10:**
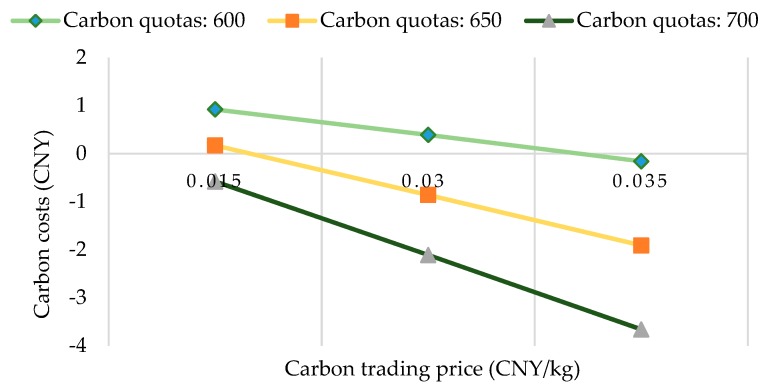
The carbon costs under different carbon trading price and carbon quotas.

At last, in order to further study the impact of carbon trading on the objective function and carbon emission, we add two new carbon prices 0.015 and 0.035 based on the study of fixed carbon prices 0.025. Thus, a further study of changing carbon prices and carbon quotas is formed. The computational results of the changing carbon prices and carbon quotas are shown in Table 11. Figure 9 shows the changing trend of total costs under different carbon trading price and carbon quotas and Figure 10 shows the changing trend of carbon costs under different carbon trading price and carbon quotas.

From the results in Table 11, Figure 9 and Figure 10, we can obtain the following the findings:

(1) When the carbon trading prices are 0.015, 0.025 and 0.035, the corresponding carbon emissions are also fixed at 661.27, 615.50 and 595.42, respectively. This further validates that for a fixed carbon trading price, carbon emissions and optimal distribution paths will not change when the carbon quota changes. As the carbon trading prices increase, the carbon emission decrease. It also further validates that carbon trading prices directly affect carbon emissions.
(2) When the carbon trading prices and carbon quotas increase within a certain range, the total costs and carbon costs have the downward trend. When the carbon trading price is 0.035 and the carbon quotas are 700, the total costs have the lowest value 1681.53 and the carbon costs have the lowest value −3.66.

### 5.5. Discussion and Analysis

In this study, the proposed PSO-TS algorithm is proven to be more applicable to small-scale problems (pro01, pro02 and pro07), which can get the better solution quickly by this method. For the small-scale MDOVRPTW, we study the impact of carbon trading on total costs, carbon costs and carbon emissions from both carbon trading prices and carbon quotas. The main summings-up are listed as follows:

(1) When the carbon trading prices exist, the carbon costs, fuel costs and total costs are all lower and the carbon emissions are also effectively improved compared with the trading prices as zero.
(2) When the carbon quota is increasing and the carbon trading price is fixed, the total cost and carbon costs also increase but the carbon emissions fixed on a certain value. We propose and prove that the changed carbon quotas don’t affect the optimal distribution paths, which also validates that carbon trading prices directly affect carbon emissions.
(3) When the carbon trading prices and carbon quotas both increase within a certain range, the total costs and carbon costs are both decreasing. For the fixed carbon quotas, when the carbon trading prices increase, the carbon emissions will decrease. For the fixed carbon trading prices, when the carbon quotas increase, the carbon emissions will be a fixed value.

Based on these results, some advice to third-party logistics enterprises and the government is forthcoming.

For logistics enterprises, they use the technological means and path optimization methods to reduce carbon emissions to create low-carbon logistics. Technological means can develop new energy or use energy-saving cars. However, these require a huge investment compared to path optimization. Therefore, this research is very necessary for logistics companies. It can quickly optimize the distribution route for government-specific carbon trading policies. According to this study, the logistics enterprises can also take some optimization measures to balance economic costs, energy costs and environmental costs. First, they should have the awareness of environmental protection and introduce the carbon emissions into the distribution path optimization. Secondly, from the operation and management level, it can reduce business operating costs and establish a good corporate image when the carbon emissions are taken into consideration. Thirdly, logistics enterprises should respond positively to the carbon trading policies, which are proposed by the government. They can choose low-carbon strategies to reduce total costs.

For the government, in order to promote low-carbon development, they can use carbon trading policies to reach the goal of fewer carbon emissions. Firstly, the government should encourage the enterprises to save energy and reduce carbon emissions. Secondly, from the management insight, they should form a relatively complete carbon trading policies and strengthen the regulation of carbon emissions. Research shows that the government can increase carbon prices in a certain range to reduce carbon emissions. The government can give logistics companies corresponding carbon quotas as much as possible to save the company’s costs without increasing carbon emissions. It can encourage the companies to respond to the carbon trading policies.

## 6. Conclusions

Due to high energy consumption and serious environmental pollution, the global climate has become warmer and warmer. The world has placed a high value on the low-carbon economy. MDOVRPTW is a complex combinatorial optimization vehicle route problem. There has been much research into its algorithm. Many scholars have published articles on low-carbon. However, there are a few articles that combine MDOVRPTW with low-carbon. We know that carbon trading is an effective means to achieve carbon reduction goals. In this paper, we study the MDOVRPTW based on carbon trading and propose the two-phase algorithm to handle this problem. Taking into account the quality and calculation time of the solution, the PSO-TS algorithm is more suitable for the small-scale cases. This paper examines the influence of carbon trading on total costs, carbon emissions costs and carbon emissions from carbon trading prices and carbon quotas.

The main results of this article are as follows: the cost of distribution companies under carbon trading is less; changes in carbon quotas cannot cause changes in carbon emissions but can cause changes in total costs and carbon emission trading costs; when carbon prices increase within a certain range, the total costs and carbon emissions of logistics and distribution companies will decrease. These findings are expected to provide decision support for managers, especially for specific third-party logistics enterprises and to formulate transportation strategies for logistics management under the government’s carbon emission restriction policy in consideration of corporate profits and new circumstances. It also provides a valuable reference and suggestions for the government’s macro-control of environmental protection and has strong practical significance. What is more, for society, fewer carbon emissions can slow down the greenhouse effect and provide a better living environment.

In this study, we proposed the effect of carbon emissions prices and carbon quotas on total costs, carbon emission trading costs and carbon emissions. But the specific optimal carbon emissions price and carbon quota need to be further researched in the future. What is more, the study in this paper only considers the single vehicle type and the decision of a single distribution company and uses the standard test library as experimental data. The parameters of this study are uncertain in the actual distribution environment. All these make some conclusions a certain gap from practical application. In future research, the MDOVPRTW based on carbon trading can consider multiple vehicle types and cooperative games among multiple logistics companies. In addition, real data can be used to get some more real and reliable conclusions.

## Figures and Tables

**Table 1:** Description of symbols.

Symbols	Description
C	Set of customers (C=d+1, d+2, …, d+C).
H	Set of vehicles ($H=1,2,\;\ldots ,\;h_{d}$).
H′	Number of vehicles actually used.
Q	Weight of vehicle itself.
$Q_{0}$	Maximum load capacity of vehicle.
*L*	Longest length of each route.
*r*‘	Fuel consumption rate in per unit distance while vehicle is at full load.
*r*	Fuel consumption rate in per unit distance while vehicle is empty.
η	Conversion factor value of fuel consumption and carbon dioxide.
$C_{v}$	Driver salary costs of per unit vehicle.
$C_{p1}$	Waiting time costs of per unit time while vehicle reaches the customer ahead of
	the required time.
$C_{p2}$	Punishing time costs of per unit time while vehicle is behind the required time
	for the customer.
$C_{f}$	Costs of per unit fuel consumption.
$C_{ctp}$	Carbon trading price, which can be the income price for the excess carbon emissions
	quotas or the subsidy price for the inadequate carbon emissions quota.
$s_{i}$	Service time of customer *i*.
$c_{ij}$	Time or distance between node *i* and node *j*.
$T_{i}^{h}$	Actual time while vehicle reaches the customer *i*.
$\left[ET_{i},\;LT_{i}\right]$	Time window which the customer *i* requested to be satisfied.
$q_{i}$	Demand of customer *i*.
$Q_{i}^{\prime }$	Weight of goods when vehicle visited customer *i*.
$F_{fuel}$	Total amount of fuel consumption.
$T_{q}$	Amount of carbon emissions quotas which are controlled by the government.
$x_{ij}^{dh}$	If the vehicle *h* of depot *d* visits customer *j* from customer *i*, $x_{ij}^{h}$ is 1. Otherwise, $x_{ij}^{h}$ is 0.

**Table 2:** Coefficients related to carbon dioxide emissions [37].

	Fuel Consumption	Average Fuel
**Basic data**	Value of fuel heating	35.4 MJ/kg
	Factor of conversion	1 TJ = 106 MJ
**Emission factor**	Coefficient of carbon emission	74,100 kg CO_2_/TJ
	Type of fuel	Diesel

**Table 3:** The information about the customers’ demand and time.

Customer	1	2	3	4	5	6	7	8	9	10
Demand	10	5	9	15	17	8	4	9	10	4
Time	3	4	3	5	1	4	4	3	2	2

**Table 4:** Data about the test instances.

Case	*C*	*D*	*H*	*Q_0_*	*L*
pro01	48	4	8	200	500
pro02	96	4	12	195	480
pro03	144	4	16	150	460
pro04	192	4	20	185	440
pro05	240	4	24	180	420
pro06	288	4	28	175	400
pro07	72	6	12	200	500
pro08	144	6	18	190	475
pro09	216	6	24	180	450
Pro10	288	6	30	170	425

**Table 5:** Parameters related to the objective function.

Parameter	Value
*C_v_*	800 CNY/day
*C_p1_*	12 CNY /h
*C_p2_*	120 CNY /h
*r*	0.165 L/km
*r’*	0.377 L/km
*C_f_*	7 CNY /L

**Table 6:** Parameters related to PSO-TS algorithm.

Parameter	Value	Parameter	Value
*C*_1_	1.5	*C*_2_	1.5
*VarMin*	0	*VarMax*	1
*W*	0.7	*nPop*	20
*R*_1_, *R*_2_	Rand (*VarSize*)	*MaxIt*	2000

**Table 7:** The computational process of the PSO and PSO-TS.

Case	PSO			PSO-TS			Optimization Rate (%)
	Total Costs	Length of Route	Carbon Emissions	Total Costs	Length of Route	Carbon Emissions	
pro01	3594.84	2123.20	1183.22	1762.68	1100.06	615.50	50.97%
pro02	8711.70	4280.86	2369.38	4462.85	2907.05	1411.18	48.77%
pro03	19,829.61	8151.34	4457.84	15,869.19	6748.95	3673.09	19.97%
pro04	26,828.33	10,415.19	5740.98	20,745.65	8801.75	4786.78	22.67%
pro05	29,219.09	11,477.84	6445.99	21,812.08	9318.71	5231.30	25.35%
pro06	43,105.48	15,244.03	8331.37	28,002.73	11,457.53	6262.51	35.04%
pro07	6264.59	3789.37	2091.83	3426.20	2166.45	1208.70	45.31%
pro08	14,701.26	7548.94	4276.92	9635.77	5695.13	3108.81	34.46%
pro09	28,203.35	12,064.30	28,203.35	17,729.27	9012.07	4902.94	37.14%
Pro10	42,958.36	16,618.96	42,958.36	29,053.09	9012.07	6928.65	32.37%
Average	-	-	-	-	-	-	35.21%

“-”: it is not necessary to calculate these averages.

**Table 8:** The computational results of pro01-pro10 with the PSO-TS.

Case	Vehicles’ Number	Drivers’ Salary	Penalty Costs	Fuel Costs	Carbon Costs	Total Costs	Carbon Emissions	Length of Route
pro01	6	0	134.19	1613.35	15.39	1762.68	615.50	1100.06
pro02	12	0	209.04	3681.82	35.28	3926.14	1411.18	2607.05
pro03	23	5600	401.07	9776.30	91.82	15,869.19	3673.09	6748.95
pro04	29	7200	685.52	12,740.47	119.66	20,745.65	4786.78	8801.75
pro05	33	7200	557.70	13,923.6	130.78	21,812.08	5231.30	9318.71
pro06	41	10,400	777.87	16,668.30	156.56	28,002.73	6262.51	11,457.53
pro07	11	0	333.61	3056.96	30.22	3420.79	1208.70	2166.45
pro08	18	800	483.65	8274.40	77.72	9635.77	3108.81	5695.13
pro09	29	4000	557.04	13,049.66	122.57	17,729.27	4902.94	9012.07
pro10	42	9600	838.60	18,441.27	173.22	29,053.09	6928.65	9012.07

**Table 9:** The results of the comparative test when carbon trading price is 0 and 0.025.

Case	Carbon Trading Price (NCY/kg)	Carbon Costs	Fuel Costs	Total Costs	Carbon Emissions (kg)
pro01	0	0	1681.92	1803.68	631.92
	0.025	15.39	1613.35	1762.68	615.50
pro02	0	0	4085.03	4294.67	1534.80
	0.025	35.28	3681.82	3926.14	1411.18
Pro07	0	0	3274.79	3482.00	1230.38
	0.025	30.22	3056.96	3420.79	1208.70

**Table 10:** The results of the comparative test when carbon quotas are different.

Case	$T_{q}^{\prime }\;(\mathrm{kg})$	Total Costs	Carbon Costs	Carbon Emissions (CE)	$T_{q}^{\prime }-\mathrm{CE}$
pro01	600	1747.69	0.39	615.50	−15.5
	650	1746.44	−0.86	615.50	34.5
	700	1745.19	−2.11	615.50	84.5
pro02	1400	3927.14	0.28	1411.18	−11.18
	1450	3925.89	−0.97	1411.18	38.82
	1500	3924.64	−2.22	1411.18	88.82
pro07	1200	3390.79	0.22	1208.70	−8.7
	1250	3389.54	−1.03	1208.70	41.3
	1300	3388.29	−2.28	1208.70	91.3

**Table 11:** The results of the changing carbon prices and carbon quotas.

Carbon Trading Price (CNY/kg)	$T_{q}^{\prime }\;(\mathrm{kg})$	Total Costs	Carbon Costs	Carbon Emissions
0.015	600	1868.95	0.92	661.27
	650	1868.20	0.17	661.27
	700	1867.45	−0.58	661.27
0.025	600	1747.69	0.39	615.50
	650	1746.44	−0.86	615.50
	700	1745.19	−2.11	615.50
0.035	600	1685.03	−0.16	595.42
	650	1683.28	−1.91	595.42
	700	1681.53	−3.66	595.42
